# Radiological Staging of Thyroid-Associated Ophthalmopathy: Comparison of T1 Mapping with Conventional MRI

**DOI:** 10.1155/2020/2575710

**Published:** 2020-10-22

**Authors:** Lu Chen, Wen Chen, Huan-Huan Chen, Qian Wu, Xiao-Quan Xu, Hao Hu, Fei-Yun Wu

**Affiliations:** ^1^Department of Radiology, The First Affiliated Hospital of Nanjing Medical University, Nanjing, China; ^2^Department of Endocrinology, The First Affiliated Hospital of Nanjing Medical University, Nanjing, China

## Abstract

**Background:**

Accurate staging of patients with thyroid-associated ophthalmopathy (TAO) is crucial for clinical decision. Full cognition of pathologic changes and staging TAO using conventional T2-weighted imaging is still limited.

**Purpose:**

To investigate the feasibility of using T1 mapping to evaluate changes of extraocular muscles (EOMs) in TAO patients, as well as to compare T1 mapping and conventional T2-weighted imaging in staging TAO.

**Materials and Methods:**

Forty TAO patients were retrospectively enrolled. “Hot spot” and “cold spot” T1 relaxation times (T1RT_HS_ and T1RT_CS_) of EOMs, as well as conventionally applied highest signal intensity ratio (SIR) of EOMs, were measured and compared between active and inactive groups.

**Results:**

T1RT_CS_ and SIR were significantly higher in active TAOs than in the inactive ones (*P* < 0.001), while T1RT_HS_ was not (*P*=0.093). Meanwhile, T1RT_CS_ and SIR were positively correlated with clinical activity score (*r* = 0.489, 0.540; *P* < 0.001). TIRT_CS_ and SIR showed no significant area under curve for staging TAO (0.830 vs. 0.852; *P*=0.748). T1RT_CS_ ≥ 1000 alone showed optimal staging specificity (90.0%), while integration of T1RT_CS_ ≥ 1000 and SIR ≥ 2.9 demonstrated optimal staging efficiency and sensitivity (area under curve, 0.900; sensitivity, 86.0%).

**Conclusions:**

Our findings suggest that the T1-mapping technique holds the potency to be utilized in TAO. The derived T1RT_CS_ of EOMs, which may be associated with fat infiltration, could be a useful biomarker to stage the disease, serving added efficiency, sensitivity, and specificity to single usage of conventional SIR.

## 1. Introduction

Thyroid-associated ophthalmopathy (TAO), also known as Graves' ophthalmopathy, is a debilitating autoimmune inflammatory disease [1, 2]. Remodeling of the orbit and upper face usually leads to series of complaints, including photophobia, grittiness, proptosis, eyelid retracting, and even vision loss [1–5]. The course of the disease can be divided into two distinct phases: active inflammation and inactive fibrosis [6]. Patients in the initially active phase is featured with mononuclear cell infiltration and edema of the orbital tissues, manifesting as redness and swelling of the eyelids and conjunctiva [2–4], and can benefit from treatments such as steroids, radiotherapy, and cyclosporine [7]. Those in the subsequently inactive phase are characterized by interstitial fibrosis, collagen deposition, and fat infiltration, with surgical decompression being the only solution [8–10]. Thus, accurate discrimination of the two phases is of significant importance in clinical practice since timely and precise treatment would facilitate efficient improvement of the disease, relieve the complaints, and limit the progression to chronic fibrotic sequelae [11].

Given the high resolution of soft tissues and absence of ionizing radiation, magnetic resonance imaging (MRI) has been increasingly applied for diagnosing and further staging patients with TAO [12, 13]. Both diameters and signal intensity ratios (SIRs) of orbital tissues have been reported to help diagnosis of TAO, while regarding staging the disease [12, 14–16], the signal intensity ratio (SIR) of extraocular muscles (EOMs) generated from fat-suppressed (FS) T2-weighted imaging (T2WI) is currently the most recognized biomarker [12, 14, 15]. However, the semiquantitative measurement of SIR, varied depending on the choice of referenced denominators, may confine the cognition of more detailed pathologic information within tissues and subsequently reduce the staging performance [17–19].

T1-mapping technology can detect tissue relaxation value in vivo and reflect different tissue properties noninvasively [20–23]. Previously, the technique has already been applied to assess various pathophysiological processes, such as chronic myocardial infarction, liver cirrhosis, renal fibrosis, and osteoarthritis [20–24], due to the superior ability of quantifying tissue characteristics of edema, fibrosis, and fat infiltration. However, concerning the utilization in orbit, knowledge is still finite [23, 24]. We suspected that the similar histopathologic changes of orbital tissues in TAO could also be detected by T1RT derived from T1 mapping, thus potentially assisting in discriminating disease phases.

Therefore, concerning the novel application of the T1-mapping technique in orbit, the aim of the present study was to explore the feasibility of using the technique to assess changes of EOMs in patients with TAO, meanwhile, to compare T1 mapping and conventional MRI in staging TAO.

## 2. Materials and Methods

### 2.1. Patients

This study was approved by our institutional review board, and written informed consent was waived due to its retrospective nature. From April 2019 to May 2020, 40 consecutive patients (mean age, 42.9 ± 14.5 years; male/female ratio, 16/24) with clinically diagnosed TAO using Bartley's criteria [25] were enrolled. The inclusion criteria included (1) both coronal T1 mapping and coronal T2WI with fat suppression were scanned before treatment; (2) image quality was adequate for further analysis; (3) no history of radiotherapy or surgical decompression; and (4) no other orbital pathologies.

Disease activity was determined for each eye according to the modified seven-point clinical activity score (CAS): spontaneous retrobulbar pain, pain on attempted up or down gaze, redness of the eyelids, redness of the conjunctiva, swelling of the eyelids, inﬂammation of the caruncle, and/or plica and conjunctival edema [25]. A CAS ≥3 was defined as active TAO, otherwise inactive. Finally, a total of 50 eyes were classified as active and 30 eyes as inactive.

### 2.2. MR Protocol

All the participants were scanned using a 3.0 T MRI system (MAGNETOM Skyra, Siemens Healthcare, Erlangen, Germany) with a 20-channel head coil. Each participant was instructed to lie still in the supine position and look at a fixed site with both eyes closed in order to reduce eye movement. Coronal T1 mapping was obtained by using a dual-flip angle-based fast spin-echo sequence. The parameters were as follows: repetition time/echo time, 4.96/1.81 ms; flip angle_1_/flip angle_2_, 2°/10°; field of view, 20 cm; slice thickness, 4.0 mm; and slice, 20. The scan time for T1 mapping was one minute and 11 seconds. Conventional structural imaging protocols included axial T1-weighted imaging (repetition time/echo time, 635/6.7 ms) and axial, coronal, and sagittal T2WI with fat suppression (repetition time/echo time, 4000/75–117 ms). The other parameters for coronal T2WI with fat suppression were as follows: field of view, 18 cm; slice thickness, 3.5 mm; and slices, 18.

### 2.3. Image Analysis

**Figure 1:**
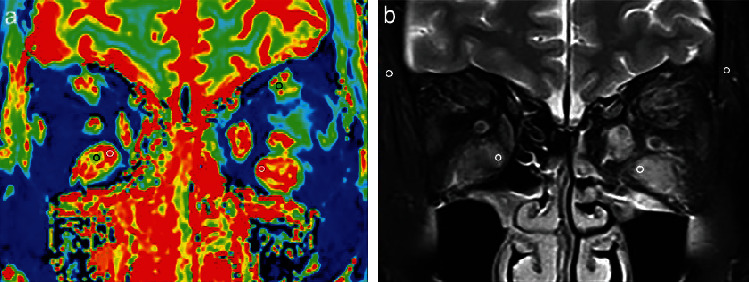
Methods for measurements of T1RT_HS_, T1RT_CS_, and SIR: coronal T1 mapping (a) and coronal fat-suppressed T2-weighted imaging (b) in a 52-year-old male with active TAO. Two circular regions of interest measuring 5–10 mm^2^ were placed in the area with the highest (white) and lowest (black) signal intensity by naked eye, representing T1RT_HS_ and T1RT_CS_, respectively (a). Meanwhile, two similar-sized regions of interest were manually placed in the area of the most inflamed EOM with the highest signal intensity as well as ipsilateral temporal muscle (b). T1RT_HS_, T1RT_CS_, and SIR values of the left/right eye were 2426/2006 ms, 1365/1422 ms, and 4.14/3.95, respectively. T1RT = T1 relaxation time; HS = hot spot; CS = cold spot; SIR = signal intensity ratio; TAO = thyroid-associated ophthalmopathy.

Imaging data of coronal T1 mapping and coronal T2WI with fat suppression were evaluated by applying a standalone platform (syngo.via, Siemens Healthcare, Erlangen, Germany). Quantitative measurements over four EOMs, including superior, inferior, medial, and lateral recti were performed in each unit of eye. On the T1-mapping image, two circular ROIs (measuring 5–10 mm^2^) were manually drawn in the area with the highest and lowest degree of signal intensity (SI) over four EOMs observed by the naked eye, representing “hot spot” (HS) and “cold spot” (CS), respectively (Figure 1(a)). Once the regions of interest were determined, the mean values of T1RT_HS_ and T1RT_CS_ were automatically generated and recorded. Meanwhile, two similar-sized regions of interest were manually placed in the area of the most inflamed EOM with the highest SI as well as ipsilateral temporal muscle on T2WI with fat suppression (Figure 1(b)). Then, the SIR value was calculated by using the formula: SI_EOM_/SI_ipsilateral temporal muscle_ [26].

Two independent radiologists (observer 1: with 9 years of experience in head and neck radiology and observer 2: with 4 years of experience in head and neck radiology), who were blinded to study design, acquisition parameters, and clinical information, independently placed the regions of interest. The measurement results of these two observers were used to assess the interobserver agreement, and the measurement was repeated by observer 1 with a washout period of at least 1 month, in order to evaluate the intraobserver reproducibility.

### 2.4. Statistical Analysis

All numeric data were averaged and reported as mean ± standard deviation. Kolmogorov–Smirnov test was used for analyzing the normality. Clinical variables (e.g., sex, age, smoking history, and CAS) were collected and compared between active and inactive groups. The differences of T1RT_HS_ and T1RT_CS_ between active and inactive TAOs were compared using Mann–Whitney *U* test. Independent sample *t*-test was applied for the comparison of SIR between two groups. The spearman correlation was used to identify the correlations between significant MRI parameters and CAS. Analyses of receiver operating characteristic (ROC) curves were performed to evaluate the staging performance of significant parameters. Comparisons of multiple ROC curves according to DeLong et al. [27] were used to compare the diagnostic performances between different techniques.

Inter- and intraobserver agreements of quantitative parameters were accessed by the intraclass correlation coefficient (ICC). The ICC values range between 0 and 1.00, and values closer to 1.00 represent better reproducibility. They were interpreted as follows: <0.40, poor; 0.41–0.60, moderate; 0.61–0.80, good; and ≥0.81, excellent. All statistical analyses were carried out with SPSS software package (v. 23.0; IBM, Armonk, NY). A two-sided *P* value less than 0.05 was considered statistically significant.

## 3. Results

There were no significant differences on age (43.5 ± 13.5 vs. 41.9 ± 16.5, *P* = 0.586), sex distribution (male/female, 10/14 vs. 6/10, *P*=0.792), and smoking history (11/25 vs. 6/15, *P*=0.804) between active and inactive TAOs. The CAS of the active group was significantly higher than that of the inactive group (3.9 ± 0.6 vs. 1.5 ± 0.5, *P* < 0.001).

**Figure 2:**
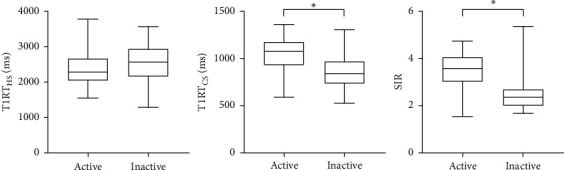
Box-plot showing the comparisons of T1RT values and SIR between groups. An asterisk indicates a significant difference (^*∗*^*P* < 0.001).

Both T1RT_CS_ and SIR were significantly higher in active TAOs than inactive ones (T1RT_CS_: 1063.72 ± 164.27 vs. 852.57 ± 165.45, *P* < 0.001; SIR: 3.46 ± 0.74 vs. 2.46 ± 0.70, *P* < 0.001), while T1RT_HS_ was not (2369.02 ± 415.92 vs. 2539.47 ± 523.44, *P*=0.093) (Figure 2). T1RT_CS_ and SIR were positively correlated with CAS (*r* = 0.489, 0.540; *P* < 0.001). Good to excellent intra- and interobserver reproducibility were obtained during the measurements of T1RT_HS_, T1RT_CS_, and SIR (ICC ranging from 0.745 to 0.911).

**Figure 3:**
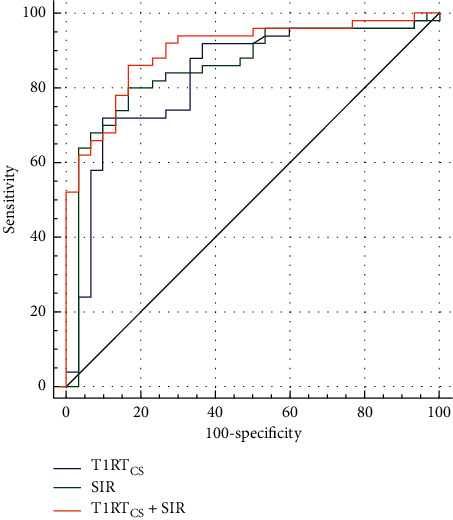
Receiver operating characteristic curves of significant parameters for staging TAO.

**Figure 4:**
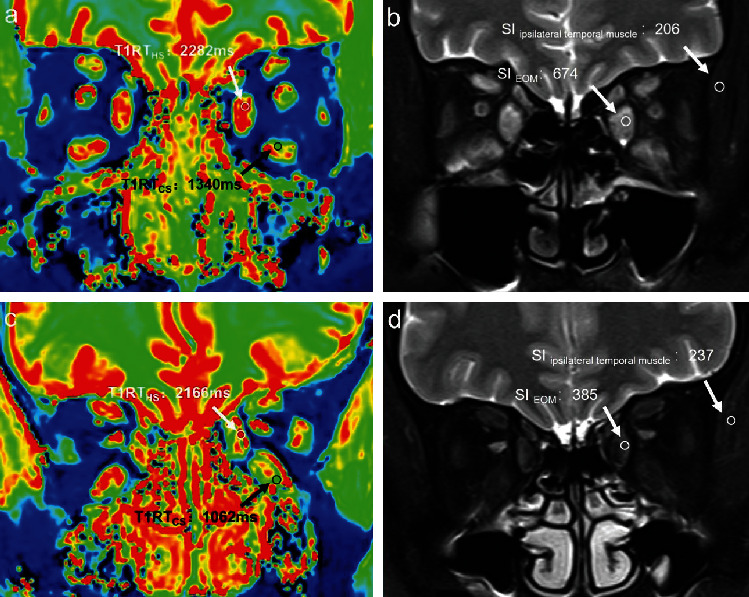
A 33-year-old male with active TAO (a, b) and a 54-year-old female with inactive TAO (c, d) were presented. Taken the left eye for example, the values of T1RT_HS_, T1RT_CS_, and SIR were 2282 ms, 1340 ms, and 3.27 for the patient with active TAO (a, b) and 2166 ms, 1062 ms, and 1.62 for the patient with inactive TAO, respectively (c, d).

Detailed ROC analyses results are displayed in Table 1. Setting cutoff values of 1000 for T1RT_CS_ or 2.9 for SIR, respectively, optimal discriminating efficiency between active and inactive TAOs of single variable was obtained, without significance in area under curve (0.830 vs. 0.852, *P*=0.748). T1RT_CS_ ≥ 1000 alone showed optimal staging specificity (90.0%). When combining T1RT_CS_ ≥ 1000 and SIR ≥ 2.9, optimal staging efficiency and sensitivity could be achieved (area under curve, 0.900; sensitivity, 86.0%) (Figure 3). Two representative cases of active and inactive TAOs are presented in Figure 4.

## 4. Discussion

T1-mapping technique has been increasingly employed to assess various diseases, whereas the data regarding its application in orbital diseases are still lacking. Considering the clinical significance for accurate staging TAO, we innovatively measured both T1RT_HS_ and T1RT_CS_ in this study, in order to acquire exhaustive information of the microstructural change within involved EOMs, as well as full demonstration of the competence of the technique in discriminating TAO phases. Our results proved the potency of T1-mapping technique in characterizing changes of orbital tissues, and in particular, they highlighted the usefulness of T1RT_CS_ in improving staging performance of TAO.

We discovered that T1RT_CS_, representing the lowest SI on T1 mapping in EOMs, was significantly higher in active TAO patients than the inactive ones. Previously, Bouazizi K et al. declared that fat infiltration in myocardium presented lower SI on T1 mapping with reduced T1RT [28]. In the domain of orbit, the only related study focused on refractory diplopia also suggested that low T1RT in EOMs, probably associated with fat infiltration, may be useful to predict response to intravenous glucocorticoid-resistant diplopia [24]. In accordance with prior findings, it is of no surprise that inactive TAOs in our cohort, with one of the main features being fat infiltration, would have lower T1RT_CS_ than active ones. The positive correlation between T1RT_CS_ and CAS further indicates that the degree of fat infiltration might increase accompanied with disease inactivity.

Referring to the other variables of T1RT_HS_, which represent the highest SI on T1 mapping in EOMs, no significance was observed between groups. Native T1RT is apt to increase both water content and fibrosis component [24, 29]. Thus, considering that T1RT_HS_ in the active phase dominated by water and that in inactive mimics dominated by fibrosis would share high values, and the nonsignificant between-group result could be reasonable.

In this study, originally applied SIR of EOMs undoubtedly increased in the active TAO group, which is compatible with previous studies, connected with inflammatory change in tissues [12, 29, 30]. However, ROC curve results indicated that single application of SIR gained no impressive staging sensitivity (80.0%) and specificity (83.3%). We found that T1 mapping could supply added staging performance to SIR alone. Using T1RT_CS_ alone could achieve optimal discriminating specificity (90.0%) between active and inactive phases, and integration of T1RT_CS_ and SIR enabled improved discriminating efficiency (0.900) and sensitivity (86.0%).

Previously, most of the imaging studies focused on the pathological changes of inflammatory edema within EOMs using the T2WI-related technique [12, 14]. However, besides the inflammatory edema, fat infiltration was also a crucial pathological feature in the EOMs of TAO patients [24]. In the current study, we tried to use the T1-mapping technique to correspond with the potential change of fat infiltration, and positive results were obtained. New metrics associated with a new aspect of the pathological changes in the EOMs of TAO patients could provide additional information besides inflammatory edema, and naturally, the diagnostic performance was improved. Our study provided a novel and promising approach to improve the current discrimination level of disease phases and would build more confidence to clinicians in selecting individual treatment strategy.

Our study has several limitations. First, this is a retrospective study with relatively small sample size and hence may have a selection bias. The nonsignificant difference of T1RT_HS_ between the active and inactive groups might hold the potential to be influenced by the limited sample size. Therefore, a large sample study is suggested to be conducted to verify our results. Second, histological analysis of EOMs was difficult to be available. Therefore, exact pathological state of EOMs is still unclear. Future study clarifying the association between the imaging parameters and the pathological changes of EOMs would be more valuable. Third, only native T1RTs were measured. Furthermore, T1 mapping with gadolinium-enhancement may help discrimination of water and fibrosis contents, thus supplying more optimized staging performance.

In conclusion, our study indicated that the T1-mapping technique is potential to be generalized in assessing TAO. The derived T1RT_CS_ of EOMs, probably associated with fat infiltration, may be a useful biomarker which could provide added performance to conventional SIR alone for staging TAO patients.

## Figures and Tables

**Table 1:** Discriminating performance of T1RT_CS_ and SIR between active and inactive TAOs.

Parameters	AUC	Cutoff	Sensitivity %	Specificity %
T1RT_CS_	0.830 (0.732–0.928)	1000.00	72.0(57.5–83.8)	90.0(73.5–97.9)
SIR	0.852 (0.761–0.943)	2.90	80.0(66.3–90.0)	83.3(65.3–94.4)
T1RT_CS_ + SIR	0.900 (0.832–0.968)	—	86.0(73.3–94.2)	83.3(65.3–94.4)

The unit of T1RT_CS_ is ms. T1RT = T1 relaxation time; CS = cold spot; SIR = signal intensity ratio; TAO = thyroid-associated ophthalmopathy; AUC = area under curve.

## Data Availability

The data used to support the findings of this study are available from the corresponding author upon request.
